# The Revolution of Digital Therapeutics (DTx) in the Pharmaceutical Industry and Their Quality Impacts

**DOI:** 10.7759/cureus.66792

**Published:** 2024-08-13

**Authors:** Anuciya Rajendran, Alekhya Kella, Damodharan Narayanasamy

**Affiliations:** 1 Department of Pharmaceutical Quality Assurance, SRM College of Pharmacy, Faculty of Medicine and Health Science, SRM Institute of Science and Technology, Chengalpattu, IND

**Keywords:** digital health technologies (dht), digital therapeutics (dtx), software systems, medical device, digital technologies

## Abstract

An increasing number of developments and trends are driving the expansion of the digital therapeutics (DTx) market in the pharmaceutical industry. Digital therapeutics are therapies intended to treat, diagnose, and prevent diseases by using patient-directed clinically assessed software applications, which can optimize the effectiveness and delivery of healthcare. These digital innovations became important as the world changed, particularly during the coronavirus pandemic. Nowadays pharma companies are getting more comfortable with the idea of digital therapies. The majority of pharmaceutical companies are examining how to incorporate pharmaceuticals and digital therapies into their treatment regimens, leveraging digital tools to enhance patient outcomes and streamline healthcare delivery. A thorough overview of the most recent technological advancements in the creation of digital therapies shows particular technologies that are essential to the market's future growth. Moreover, the evaluation of digital therapeutics by clinical trial and real-world data is outlined. The critical quality attributes of DTx products and the challenges, including data management issues and regulatory obstacles, which make the creation, approval, and marketing of customized medicines more difficult, are covered in this review article. Overall, pharma companies are venturing into the world of digital therapeutics while acknowledging the limitations of the emerging field.

## Introduction and background

According to the Digital Therapeutics Alliance (DTA), “digital therapeutics provide evidence-based therapeutic interventions to patients by means of qualified software programs to prevent, manage, or treat medical conditions. They can be used as monotherapy or in combination with other therapies, including medications or devices to improve outcomes” [1]. It is the integration of digital technologies, such as telemedicine, mobile apps, genomics, robotics, and artificial intelligence, into the healthcare system to increase the effectiveness of healthcare delivery. In the modern era, these digital therapeutics products, either under development or in the market, are widely applied to treat medical conditions like neuropsychiatric disorders, cancer management, and diabetes treatment [1].

Notably, a number of concepts like telemedicine, mHealth (mobile health), and eHealth (electronic health) have gained traction in the context of digital health technology. The term “eHealth” refers to the application of communications and information technology to promote health. The term “mHealth” refers to the use of wireless mobile technology for public health due to the increasing popularity and widespread use of smartphones and related apps. The term “telemedicine” is applied to the practice of providing medical treatments remotely via electronic communications and information technology [2]. For example, a contemporary topic that occupied the attention of the scientific and healthcare community and the government, especially during the COVID-19 global pandemic, was that most people all over the world suffered in one way or another from home quarantines, communal lockdowns, and limited access to healthcare. The COVID-19 pandemic, which hit the world in early 2020, accelerated the advancement and use of both novel and well-established digital health technologies, such as telehealth and wellness apps. Regulatory approval is necessary for digital medicine products that fall under the category of "medical devices". Some examples of such software include decision-support software, software for remote patient monitoring, and digital diagnostics. This was accomplished through increasing financing, hastening policy approval, enhancing government priorities, creating new public-private partnerships, and planning and directing a range of collaborative research initiatives. Approximately half of all Americans experience chronic illnesses, which drive up the annual healthcare costs in the country to 86% [3]. Digital therapeutics (DTx) applications have been approved for the treatment of a variety of chronic diseases, such as type 2 diabetes, hypertension, asthma, chronic obstructive pulmonary disease (COPD), and some mental health issues, such as anxiety and depression. People with type 2 diabetes are primarily found in emerging economies such as India and China. Thus, the adoption of digital technologies in the healthcare system could serve as a driving force for improving public health in general.

## Review

Digital therapeutics

Digital therapies refer to any digital technology that provides evidence-based therapeutic treatments for the purpose of managing, treating, or preventing a disease or medical issue. DTx products are reimbursable and prescriptible, and they are produced through controlled clinical inquiry and regulatory approval for specific indications. The active principle of DTx is different from that of a traditional medication since it is made up of algorithms and software rather than a chemical or biological substance. A two-step procedure can be used to develop drug + digital combination therapies: (1) DTx was developed via the "software as a medical device" regulatory process; (2) DTx-Rx combo product was developed, even though DTx is a medical device [4]. DTx with conventional medication, sometimes referred to act as a catalyst or add-on treatment, may increase the clinical efficacy, safety, and patient compliance of the related drug treatment. A fundamental characteristic of digital therapeutics is the "optimization" of a patient's treatment plan, which brings the concept of personalized medicine to life. The difference between digital healthcare and traditional healthcare is discussed in Table 1.

**Table 1:** Difference between Digital Healthcare and Traditional Healthcare

Digital Healthcare	Traditional Healthcare
Interaction of patient and physician through machine	Direct interaction between the patient and the physician
Patients and several stakeholders share and maintain the data	Hospitals or institutions maintain the data
Customized therapy, personalized medicine with non-traditional procedures. Mass screening, asymptomatic, or early preclinic diagnosis, expected technology, and support for physician decision-making	Standardized therapy based on traditional clinical procedures and the experience of physicians. Signs and symptoms, medical testing, diagnosis, and treatment strategy
As long as the patient is there, the medical care or examination point may change	The lab or clinic serves as a location of medical care or examination
The patient actively participates in decision-making with the physician acting as a collaborator, adviser, or guide	Physicians act as key participants in diagnosis and prescribing the treatment

Digital therapeutics technologies

The four primary types of key technologies in DTx are as follows:

1. Data collection and preprocessing

2. Data analysis algorithms

3. Human-computer interaction (HCI) factors

4. Recommendation systems.

Data Collection and Preprocessing

To collect human bio-signal data, a variety of wearable sensor devices have been created, including shoe insoles, rings, bracelets, necklaces, chest bands, and smartwatch types. Heart-related data, including heart rate, heart rate variability, and ECG, are most commonly measured by photoplethysmogram sensors. Furthermore, biofluid measurement with a sweat-rate sensor and user activity detection with an accelerometer and gyroscope have demonstrated significant improvements in patient outcomes. For example, sweat-rate sensors have been used to monitor electrolyte levels and dehydration risk in athletes and patients with conditions like cystic fibrosis. Continuous monitoring of sweat composition can help in the early detection of dehydration or electrolyte imbalances, allowing for timely interventions. Accelerometers and gyroscopes in wearable devices have been employed to track physical activity and detect falls in elderly patients, enabling quicker response times and reducing the risk of injury. These applications of wearable technology contribute to more personalized and proactive healthcare. User data must be gathered without revealing personally identifiable information or it must be deidentified through additional data preprocessing steps following the data gathering stage. Pattern-matching deidentification algorithm and recurrent neural network model are the two methods for the deidentification process.

Data Analysis

The DTx service users require accurate analysis results regarding their health and highly customized remedies derived from their data. Medical AI technology such as machine learning and federated learning is used by DTx products to study and create their systems based on specific ailments using diagnostic or classification algorithms. In medical image analysis, convolutional neural network (CNN)-based methods like segmentation, augmentation, prediction, and classification are used to extract useful information and help with decision-making from the initial input image.

Human-Computer Interaction (HCI) Factors

Primary task support, system credibility support, dialogue support, and social support are the four components that make up the persuasive system design (PSD), which can promote empathy and strengthen the bond between DTx and its users. In order to improve the user's attitude, primary task support should minimize the user's participation effort while providing lots of instructions and explanations. Dialogue support must be user-friendly and give patients feedback in the form of words, pictures, symbols, or sounds. In system credibility, the service's information must be accurate and seem well-informed and experienced. It is advised to provide shared services for social support that might encourage social learning, cooperation, comparison, and acknowledgment. Digital treatments will become more effective if these PSD-based HCI components are included, as they will create a therapeutic alliance.

Recommendation System

A recommendation system (RS) that offers a customized treatment plan based on user data is closely linked to user personalization. In general, RS uses collaborative, hybrid filtering or content-based algorithms to give users customized systems. Hybrid filtering recommendation system needs to be enhanced on a constant basis by utilizing machine learning techniques to extract various aspects from the input [5].

Digital therapeutics regulations

Meeting the requirements of regulations in the development of digital health products is important and some of the regulations to be followed are the Medical Device Regulation and the Good Clinical Practices. Several ISO (The International Organization for Standardization) standards are taken into consideration during the development of digital therapeutics.

In the United States, the Food and Drug Administration (FDA) considers mobile medical applications that are intended to perform enforcement discretion and won't require manufacturers to register and disclose their applications with the FDA or file premarket review applications [6]. The DTx product "reSET-O", which is used for treating opioid use disorder, was able to successfully pass through the regulatory process by proving clinical efficacy and satisfying safety standards in the United States stipulated by the FDA.

In Europe, the European Medicines Agency, European Union, and national competent authorities (NCAs) make sure that the clinical evidence generated is scientifically valid because digital health technology tools (DHTTs) are being used to gather data and support the safety and efficacy of pharmaceuticals in clinical trials for marketing authorization applications. Where DHTTs are also a medical device, they are governed by medical device regulations and are subject to regulatory scrutiny by the appropriate device authorities (notified authorities and NCAs with device competence); these device bodies also contribute to the risk rating of medical devices [7].

The Digital Technology Assessment Criteria was introduced by the National Health Service (NHS) of the United Kingdom in February 2021. This set of criteria covers safety, technical assurance, data protection interoperability, and usability and are intended to be utilized by both digital health tool developers and NHS personnel who may eventually require them [8]. In Europe, the diabetes management app known as "mySugr" was awarded the CE (Conformité Européene) label, which ensures that it complies with the standards that govern medical devices in the European Union.

Clinical evaluation

The clinical evaluation for DTx is similar to that of pharmaceuticals and involves evaluating efficacy and safety. Preclinical evaluation, which is required for drugs, is not applicable to DTx because the latter is a software. Clinical evaluation of DTx products necessitates an integrated strategy that takes into account DTx's features such as therapeutics and medical devices [6]. The two different strategies for evaluating DTx are clinical trials and real-world data.

Evaluation by Clinical Trial

Similar to medication products, DTx products that are already FDA-approved have undergone clinical evaluation through randomized controlled trials (RCTs). The various components of a clinical trial for digital therapeutics are as follows:

1. Trial design: A parallel design or crossover design is evaluated.

2. Randomization: A stratified randomization or cluster randomization is performed.

3. Blinding: The evaluation is carried out using an open trial design; however, a sham control group might be formed and blinding can be used.

4. Participants: Participants are patients with chronic diseases, often used for preventing and managing diseases. Participants are patients with psychiatric illnesses when the DTx gets used therapeutically.

5. Control group: Control groups differ according to the device connected to the DTx, and frequently, many functionalities are removed to form a sham control group. Subdividing the functions might help construct a control group if the functions are complex.

6. Number of participants: From minimal pilot studies to extensive research involving over 1,000 individuals, the number of participants varies.

7. Adverse events: A small number of side effects associated with DTx may be reported [9].

Evaluation by Real-World Data

The term "real-world data" typically refers to data gathered from actual clinical settings. Real-world data includes information from patient registries, post-marketing surveillance, claims data, and practical clinical studies. Usage data-based clinical evaluations are anticipated to become more prevalent as these kinds of DTx have the ability to easily gather substantial volumes of usage data. For example, a number of clinical studies based on usage data have been conducted on the DTx product known as "Omada Health," which was developed for the purpose of preventing diabetes. These evaluations have demonstrated that the product is beneficial in reducing the participants' body weight and improving their health behaviors. In a similar manner, the digital therapy for insomnia known as "Big Health's Sleepio" successfully collected user engagement data during clinical trials in order to evaluate the impact of insomnia on the quality of sleep and mental health. The results of these trials have shown significant improvements in sleep outcomes.

Smarter treatment and recent advancements

A doctor and co-founder of Proteus Digital Health, George Savage, who lives in California, states that “efficacy is what a drug can do; effectiveness is how it works in the real world and right now we have a large efficacy-effectiveness gap.” The primary problem is that globally 25% to 50% of people do not take their prescription drugs as prescribed. This has been connected to 125,000 fatalities in the United States alone and is thought to cost up to $289 billion a year. These can be minimized by adopting digital therapeutics, which have sensors to monitor the activity of users and record the medication responses. “You can think of this as a digital nurse,” says Savage [10].

Currently, the majority of digital therapeutics products on the world market come in the form of proprietary software, including medical platforms or mobile applications. An app called "Hypertension Digital Therapeutics" promotes lifestyle modifications for non-pharmacological treatment components that have been shown to effectively lower blood pressure, in accordance with national and international norms [11]. According to the findings of a recent study, a digital therapeutic for hypertension, which was utilized by more than 500 patients, led to a significant reduction in systolic blood pressure by an average of 12.2 mmHg over the course of six months. This finding highlights the effectiveness of digital therapeutics in regulating blood pressure through behavioral interventions.

**Figure 1:**
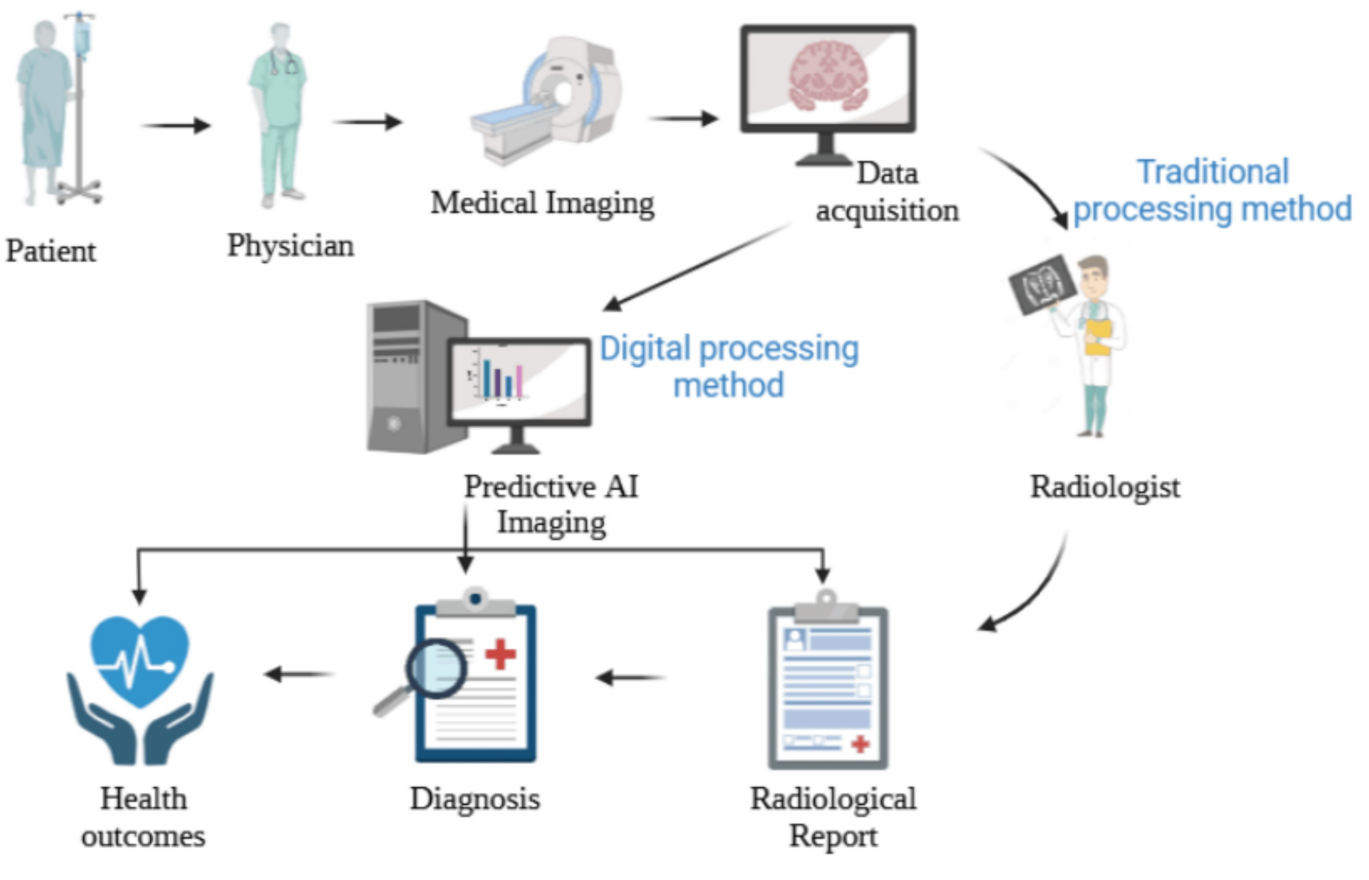
Traditional processing method and digital processing method Analysis of Biomedical Images: Artificial intelligence (AI) has the potential to streamline the diagnostic process for image specialists and yield faster health outcomes by eliminating the need for several steps in the review and diagnosis process, which is a common practice in traditional approaches. AI can assist with detecting illnesses that are sometimes hard for the human eye to perceive and diagnose, as well as identifying, classifying, and extracting pathological regions more quickly and with precision in diagnosis. From Ona, S. (2023). "Revolutionizing Our Healthcare: Harnessing AI's Potential for Health and Medicine". https://app.biorender.com/biorender-templates/t-65270cd349c5b1089b610edd-revolutionizing-our-healthcare-harnessing-ais-potential-for-

The recent advancements in DTx have quantifiably impacted patients, doctors, and caregivers’ overall experiences in receiving healthcare. Compared to the traditional method, the digital method is multiple times faster as shown in Figure 1. Digital stethoscopes with an accompanying electrocardiogram (ECG) are one of a few other digital health platforms and solutions that are advantageous to doctors and patients. These devices can be linked with electronic medical records. The advancement of experimental and computational methodologies to forecast and assess multimodal tumor therapy responses through the integration of 3D bioprinting and machine learning in a therapeutically related setting. Furthermore, the investigation of the complex tumor-immune microenvironment properties was made possible by this integrative approach [12].

Quality attributes and their impacts on digital therapeutics

Digital therapeutics are software systems. The quality and safety of these software systems have become prominent management disciplines in the healthcare industry and other sectors where safety margins are extremely low. Documentary requirements for models, designs, testing, specifications, and developed components are part of these standards.

A drug ingredient or drug product's critical quality attributes and requirements are defined in four main categories by monographs: identity, purity, strength, and performance. Each of these characteristics will be defined similarly for DTx as follows [13].

Identity

The product identity and its identification requirements must comply with standards, or it is considered to be misbranded, adulterated, or both. DTx products have unique codes that act as an identity for these products. As the functionality and mode of action remain the same, modifying the code through upgrades and/or improvements might not be regarded as a new DTx. Therefore, the code's identity actually lies in its function or output. Particularly, a DTx, its digital excipients, and its digital application programming interface (API) could be subjected to identity testing against a set of predicted inputs, outputs, and behaviors.

Strength

Quantitative tests, sometimes referred to as strength tests, are used to ascertain the concentration of an active pharmaceutical ingredient (API) in a medicinal product. The strength of DTx products can be viewed in two main ways: (1) quantity of product available to the patient and (2) frequency of patient exposure to the DTx product. There is no mutual exclusion between these two positions. Maintaining the strength and ensuring the patient gets the prescribed dose is essential when changes and upgrades are made to the DTx over its lifetime.

Purity

To determine a medicinal product's quality, it is essential to evaluate its purity and spot impurities. The critical attribute of purity acknowledges that components other than the desired active ingredients and excipients might be present. Impurities are nothing but contaminants and adulterants. The purity of DTx may be affected by (1) errors (bugs) that could cause performance issues and (2) undesired behavior, whether malevolent or benign, similar to contaminants and adulterants. Examples include unwanted code that destabilizes, degrades, or otherwise negatively affects a DTx, leaks of (meta)information to uninvited parties, and computer viruses or malware that directly affect the DTx.

Performance

According to the pharmacopeial approach, performance is the degree to which the marketed pharmaceutical product possesses the physical attributes needed to produce the desired therapeutic effect at the intended site. Demonstrating the DTx product's performance consistency over the span of its lifetime is important. The parameters of product performance consistency for DTx are efficacy, safety, user engagement and adherence, reliability, stability, data accuracy, interoperability, and patient satisfaction.

Challenges

Some commercially accessible digital health tools are unable to unequivocally show their clinical relevance and advantages for healthcare providers. Regarding the use of digital health in clinical practice and medical education, the other problem is the absence of clear clinical guidance. Another important issue is the industry's alienation from innovators, investors, and practicing healthcare professionals, as well as their lack of close communication. Some of the challenges and disruptions are the following.

Data Security and Privacy

An enormous amount of data is collected with or without including personal information by mobile health apps. Since health data is sensitive, hackers would likely target it, so security and privacy are important issues in the field of digital health [14].

Regulatory Considerations

The absence of specific standards to ensure the security of these gadgets and the caliber of the data gathered is one of the barriers to the growth of DTx. Moreover, different countries have different guidelines and regulations for DTx [15].

Public Awareness

Educating people about the advantages and disadvantages of DTx is essential, as many patients and healthcare professionals are reluctant to change and not entirely aware of the benefits of DTx.

Cost and Availability

The public demands that novel therapies be affordable and accessible, yet pharmaceutical price is still a divisive topic. Finding the ideal balance between maintaining investments in research and development and fair pricing is a constant struggle.

Flexibility and Innovation

To adjust to the changing environment, pharmaceutical businesses must embrace agility and cultivate an innovative culture. The agility to investigate novel business models and make necessary adjustments is crucial due to the swift changes in technology [16]. To address these challenges effectively, organizations can implement the following strategies: invest in continuous learning, adopt agile methodologies, implement scenario planning, and strategic partnerships. 

Revolution of digital therapeutics in pharma industry in the future

Digital medicines represent a powerful way to change treatment paradigms and design a future where patient-centered care and wellbeing are prioritized as they stay embedded in the healthcare system [17]. According to a worldwide survey by McKinsey & Company, more than 75% of patients anticipate using digital health services in the future, if those services fulfill the standard of care that they require. In the current digital health era, both larger tech corporations and smaller startups are competing to offer on-demand digital health services [17]. The tools like Apple Health Kit help to track fitness activities and nutrition, while research kits help researchers develop digital health tools for the purpose of medical research. The development of ethically acceptable digital health solutions is under more strain than ever as the sector experiences revolutionary innovation. Companies are striving to interpret enormous amounts of data in order to enhance predictive analytical methods and implement inventive approaches to enhance the standard of care and results. Future economic growth in the DTx market may match or even overtake that of traditional pharmaceuticals, leading to increased access to real-world data. Digital health is here for the long term and will alter the present status, as seen by its increasing acceptance and implementation by doctors, patients, regulators, and the healthcare sector. In the future, DTx are going to be a game-changer in the healthcare industry. Their effectiveness has been demonstrated, and they will be integrated with cutting-edge technology such as artificial intelligence and wearables, which will drive universal adoption. There will be a continued increase in investments in DTx, which will help to foster innovation and enhance their role in individualized care. As regulatory frameworks continue to develop, DTx will become more widely accepted and integrated into conventional healthcare. This will pave the way for health management systems that are more comprehensive, data-driven, and time-based.

## Conclusions

Digital therapeutics aim to make the world’s healthcare system better through providing more effective therapies, personalized treatment, and more precise diagnoses. Digital therapeutics, which combine state-of-the-art technology and clinical knowledge to provide individualized, efficient, and easily available treatments, represent a significant shift in the healthcare industry. Digital therapies have the potential to replace traditional treatments with safer and more affordable solutions, saving millions of dollars in medical costs. Moreover, the advent of the digital revolution has been gradually bringing about significant changes on all fronts in the modern economy, society, businesses, and labor market. The future of digital therapeutics hinges on the continued collaboration between pharmaceutical companies and tech firms, which will drive innovation and ensure that DTx can be safely and effectively integrated into healthcare. However, resolving integration, privacy, and regulatory issues is necessary to realize their full potential. For instance, combining pharmaceutical expertise with advanced tech solutions has already led to successful digital interventions for chronic conditions like diabetes and mental health. Digital medicines have the potential to significantly impact healthcare in the future by facilitating the development of creative and patient-centered solutions through continued breakthroughs and strategic partnerships.

## References

[1] Hong JS, Wasden C, Han DH (2021). Introduction of digital therapeutics. Comput Methods Programs Biomed.

[2] Herold F, Theobald P, Gronwald T, Rapp MA, Müller NG (2022). Going digital - a commentary on the terminology used at the intersection of physical activity and digital health. Eur Rev Aging Phys Act.

[3] Akhtar N, Sami H, Jain A, Singhai AK (2023). Digital therapeutics: an updated review. Inventi Rapid: NDDS.

[4] Biskupiak Z, Ha VV, Rohaj A, Bulaj G (2024). Digital therapeutics for improving effectiveness of pharmaceutical drugs and biological products: preclinical and clinical studies supporting development of drug + digital combination therapies for chronic diseases. J Clin Med.

[5] Yoo JH, Jeong H, Chung TM (2023). Cutting-edge technologies for digital therapeutics: a review and architecture proposals for future directions.. Appl Sci.

[6] Sverdlov O, van Dam J, Hannesdottir K, Thornton-Wells T (2018). Digital therapeutics: an integral component of digital innovation in drug development. Clin Pharmacol Ther.

[7] Colloud S, Metcalfe T, Askin S (2023). Evolving regulatory perspectives on digital health technologies for medicinal product development. NPJ Digit Med.

[8] Rassi-Cruz M, Valente F, Caniza MV (2022). Digital therapeutics and the need for regulation: how to develop products that are innovative, patient-centric and safe. Diabetol Metab Syndr.

[9] Huh KY, Oh J, Lee S, Yu KS (2022). Clinical evaluation of digital therapeutics: present and future. Healthc Inform Res.

[10] Makin S (2019). The emerging world of digital therapeutics. Nature.

[11] Kario K, Harada N, Okura A (2022). The first software as medical device of evidence-based hypertension digital therapeutics for clinical practice. Hypertens Res.

[12] Tang M, Jiang S, Huang X (2024). Integration of 3D bioprinting and multi-algorithm machine learning identified glioma susceptibilities and microenvironment characteristics. Cell Discov.

[13] Ambrose M, Seiler D, Barrett B, Yu C, Podolsky D, Levy M. (2020). The Role of Public Standards in Assuring Quality of Digital Therapeutics. USP Digital Therapeutics.

[14] Cummins N, Schuller BW (2020). Five crucial challenges in digital health. Front Digit Health.

[15] Trifiro G, Crisafulli S, Puglisi G (2021). Digital therapeutics like pharmaceuticals?. Tendenze nuove.

[16] Haider R (2023). The future of pharmaceuticals industry 2024. Journal of Pharmaceutics and Pharmacology Research.

[17] Park MPH S, Garcia-Palacios J, Cohen A (2024). Sophie Park MP, Garcia-Palacios J, Cohen A, Varga Z. From treatment to prevention: the evolution of digital healthcare (sponsor feature produced by Bayer and G4A). Nature Portfolio. Nature.

